# Fold-change of chromatin condensation in yeast is a conserved property

**DOI:** 10.1038/s41598-022-22340-8

**Published:** 2022-10-17

**Authors:** Katreena Yamin, Swati Bijlani, Judith Berman, Awakash Soni, Joseph Shlomai, Bijoy Mukut Buragohain, Michal Werbner, Meital Gal-Tanamy, Avi Matityahu, Itay Onn

**Affiliations:** 1 Chromosome Instability and Dynamics Lab, The Azrieli Faculty of Medicine, Bar-Ilan University, Safed, Israel; 2 Shmunis School of Biomedical and Cancer Research, The George S. Wise Faculty of Life Sciences, Tel Aviv University, Tel Aviv, Israel; 3 Department of Surgery, Beckman Research Institute, City of Hope National Medical Center, Duarte, CA USA; 4 Department of Microbiology and Molecular Genetics, The Institute for Medical Research Israel-Canada and the Kuvin Center for the Study of Infectious and Tropical Diseases, The Hebrew University of Jerusalem, Jerusalem, Israel; 5 Molecular Virology Lab, The Azrieli Faculty of Medicine, Bar-Ilan University, Safed, Israel

**Keywords:** Nuclear organization, Cell biology, Chromosomes, Eukaryote, Chromosomes

## Abstract

During mitosis, chromatin is condensed and organized into mitotic chromosomes. Condensation is critical for genome stability and dynamics, yet the degree of condensation is significantly different between multicellular and single-cell eukaryotes. What is less clear is whether there is a minimum degree of chromosome condensation in unicellular eukaryotes. Here, we exploited two-photon microscopy to analyze chromatin condensation in live and fixed cells, enabling studies of some organisms that are not readily amenable to genetic modification. This includes the yeasts *Saccharomyces cerevisiae, Schizosaccharomyces pombe, Kluyveromyces lactis,* and *Candida albicans*, as well as a protist *Trypanosoma brucei*. We found that mitotic chromosomes in this range of species are condensed about 1.5-fold relative to interphase chromatin. In addition, we used two-photon microscopy to reveal that chromatin reorganization in interphase human hepatoma cells infected by the hepatitis C virus is decondensed compared to uninfected cells, which correlates with the previously reported viral-induced changes in chromatin dynamics. This work demonstrates the power of two-photon microscopy to analyze chromatin in a broad range of cell types and conditions, including non-model single-cell eukaryotes. We suggest that similar condensation levels are an evolutionarily conserved property in unicellular eukaryotes and important for proper chromosome segregation. Furthermore, this provides new insights into the process of chromatin condensation during mitosis in unicellular organisms as well as the response of human cells to viral infection.

## Introduction

The fidelity of chromosome segregation depends on the reorganization of interphase chromatin into compact mitotic chromosomes. This fundamental property of chromatin ensures the equal separation of the sister chromatids during anaphase. Chromosome condensation, an evolutionarily conserved property of cells, is mediated by the condensin complex, which extrudes chromatin fibers into rosettes in a tidily regulated process^1–4^. In higher eukaryotes, the morphological changes in chromatin structure between interphase and mitosis are visible due to the significant changes in condensation levels. For example, in human cells, mitotic chromosomes are about 2000-fold more compact than interphase chromatin^5,6^. However, in microorganisms, small genome and nuclear size, and low condensation levels make the direct visualization of chromosomes and the measurement of chromatin compaction levels challenging.

*Saccharomyces cerevisiae* (budding yeast) has served as an important model organism for the study of chromosome biology. Traditionally, yeast chromosome condensation is studied using mitotic chromosome spreads of lysed mitotic nuclei. The chromosomes are adhered to a glass slide, stained, and visualized by microscopy^7^. However, yeast nuclei and mitotic chromatin are difficult to study using light microscopy because their size is close to the limit of resolution. A typical image of the 16 chromosomes of a haploid yeast cell appears as a single mass with a distinctive emerging loop composed of the single rDNA locus (e.g., see^8–14^), which is commonly used as a biomarker for condensation. Light microscopy of the rDNA is labor-intensive and limited to analyzing the condensation of a single locus. Alternative assays, based on measuring the distance between distinct loci on a single chromosome, are not being used extensively^14,15^. Furthermore, all of these methods have been developed for studies of *S. cerevisiae*, but not all microbial eukaryotes have a distinctive rDNA loop that can be used as a biomarker for condensation. Thus, adapting current approaches to the study of chromosome biology in distantly related species is arduous and depends on the availability of molecular tools for the detection of specific chromosomal regions.

Recently, we developed two-photon microscopy to induce second harmonic generation (SHG) in chromatin to measure condensation in live yeast^16^. SHG is a physical phenomenon in which excitation of isotropic molecules, such as chromatin with two consecutive photons, results in autofluorescence, with fluorescence emissions being proportional to the microstructure of the molecule, such that differences in chromatin condensation levels are detectable^17,18^. Using this method, we characterized changes in *S. cerevisiae* chromatin condensation as a function of the cell cycle phase or a response to genetic manipulations of cohesin or condensin. We found that mitotic chromosomes in *S. cerevisiae* are ~ 1.5-fold more compact than interphase chromatin, which stands in agreement with previous measurements^9,14,16,19^.

A critical step for measuring chromatin width is the precise focusing of the excitation laser on the chromatin; the emission signal must be collected quickly, as excitation rapidly heats the cells and damages them. To overcome the challenge of achieving accurate focus on chromatin in live cells with unlabeled nuclei, we focused on a fluorescently-tagged histone as a marker^16^. This required the expression of foreign DNA constructs in cells and thus the ability to perform DNA transformation. This process is complex, time-consuming, and not feasible with many non-model organisms, which was a limitation of the method.

Here, we expanded the repertoire of species and cell types for which two-photon microscopy can be used to study chromosome organization, and we developed methods to measure chromatin condensation in fixed, as well as live, cells. We modified the technique to accommodate fixed cells in which the chromosomes and chromatin DNA can be stained rather than detected with recombinant fluorescent proteins. We compared measurements in fixed *S. cerevisiae* cells relative to prior measurements in live cells and found that the fold change between mitosis and interphase is similar. Furthermore, we extended the analysis to other species and found that the degree of compaction is evolutionarily conserved between yeasts and trypanosomes. Finally, we demonstrated that the infection of human hepatoma cells with the hepatitis C virus (HCV) induces interphase chromatin decondensation. Thus, the two-photon microscopy approach to measuring chromatin condensation in microbial eukaryotes works across a broad swath of diverse organisms and reveals new information on chromatin dynamics.

## Materials and methods

### Yeast strains and growth

Yeast strains are listed in Supplementary Table S1. Cells (*S. cerevisiae, S. pombe, K. lactis, C. albicans*) were grown in YPD supplemented with 2% glucose at 30 °C. For repression of pTETR-SMC2 in *C. albicans*^20^, overnight cell culture was diluted to OD_600_ 0.1 in YPD supplemented with 50 μg/ml doxycycline (Sigma-Aldrich) and grown to mid-log phase. Although SMC2 is essential for cell viability, *C. albicans* cells grown in the presence of tetracycline do not die because repression of the *tet* promoter is not complete or takes a long time to be achieved.

### Preparation of yeast cells for microscopy

Live-cell imaging is described in^16,21,22^ with the following modifications for analyzing fixed cells. Cells were grown to midlog phase and fixed in 4% paraformaldehyde (Sigma-Aldrich) for 15 min at room temperature. Cells were washed once in 0.1 M KPO_4_ 1.2 M sorbitol, placed on a polylysine adhesion slide (Electron Microscopy Sciences), and allowed to adhere for 40 min. Slides were rehydrated via three 5 min washes with 1xPBS (Biological Industries). Slides were stained with mounting medium including DAPI (Sigma-Aldrich), covered with a coverslip (Kaltek), and sealed with clear nail polish. Live cell nuclei staining was done by adding 0.02 mM Hoechst33342 (Invitrogen) to mid-log cell culture for 30 min. Cells were collected by centrifugation, washed three times with 1xPBS (Biological Industries), and placed on a polylysine adhesion slide (Electron Microscopy Sciences) covered with a coverslip (Kaltek).

### Trypanosome growth and fixation

*Trypanosoma brucei brucei* Procyclic form (PF) strain 29–13 cells^23^ were grown at 28 °C in BECK’s medium, supplemented with 10% fetal bovine serum, 15 μg/ml G418, and 50 μg/ml hygromycin. The 1 × 10^7^ cells were fixed with 6% paraformaldehyde, spread on a polylysine adhesion microscope slide (Electron Microscopy Sciences), stained with DAPI (Thermo Fisher Scientific), covered with a coverslip (Kaltek), and sealed with clear nail polish.

### HCV infection of hepatoma cells

Growth and infection of Huh-7.5 cells with HCV were carried out as described in^24^. Briefly, cells were infected with the HJ3-5 chimeric virus at an MOI of 0.1–0.5 and passaged for two weeks until approximately 100% of the cells were HCV positive, as previously described^24^. High infection levels were maintained for two weeks by adding 2% human serum to the culture medium, as previously described. HCV-infected and uninfected cells were washed twice with trypsin EDTA Solution B (Biological Industries), spread onto a polylysine adhesion microscope slide (Electron Microscopy Sciences), and incubated for two hours at 37 °C. The cells were fixed in methyl alcohol/acetone for 10 min at room temperature. The slides were then stained with mounting medium, including DAPI (Sigma-Aldrich), and covered with a coverslip (Kaltek).

### Microscopy and image analysis

All types of cells were analyzed through a Zeiss LSM 780 Confocal inverted modular microscope, using a multiphoton Chameleon Vision II laser (Chameleon laser: > 3 W, pulse width at peak: 140-fs duration, repetition rate: 80 MHz, tuning range: 690–1080 nm), with a lambda PMT being used in 8.9 nm steps to simultaneously identify all of the wavelengths in the spectrum. The slides were covered with immersion oil and visualized with × 63 objective lenses, 1.4 n/a. Focus on the chromatin was accomplished by excitation/emission at 405/498 nm and followed by image analysis. Nuclei were excited by a 140 fs pulse laser at various excitation and power levels, as follows: *S.cerevisiae, K. lactis*, and *C. albicans*: 768 nm, 0.5%; *S. pombe*: 768 nm, 0.9%; *T. brucei*: 840 nm, 0.9%; Huh-7.5: 832 nm, 0.9%, where emissions were collected between 414 and 628 nm in 8.9 nm steps. Quantitation of the integrated density and the circumference of the nucleus was carried out by using ImageJ software, as described in^16^. At least 20 cells were sampled in each experiment. Statistical difference was tested using a Student’s two-tailed t-test.

## Results

### Measuring mitotic chromosome condensation in live *S. pombe* cells

To extend the utility of two-photon microscopy beyond the analysis of chromosome condensation in live *S. cerevisiae* cells^16^, we first investigated the chromatin organization of live *S. pombe* cells. We grew yIO984, a strain expressing two fluorescently labeled proteins. Cdc11 encoded the septation initiation network scaffold protein, which localized to the spindle pole body and fused to GFP. The histone H3 gene, Hht1, which localized to nuclear chromatin, fused to mCherry. Strain yIO984 was grown to mid-log phase, and cell cycle staging of individual cells was determined based on the nuclear localization of Cdc11 (Supplementary Fig. S1).

**Figure 1:**
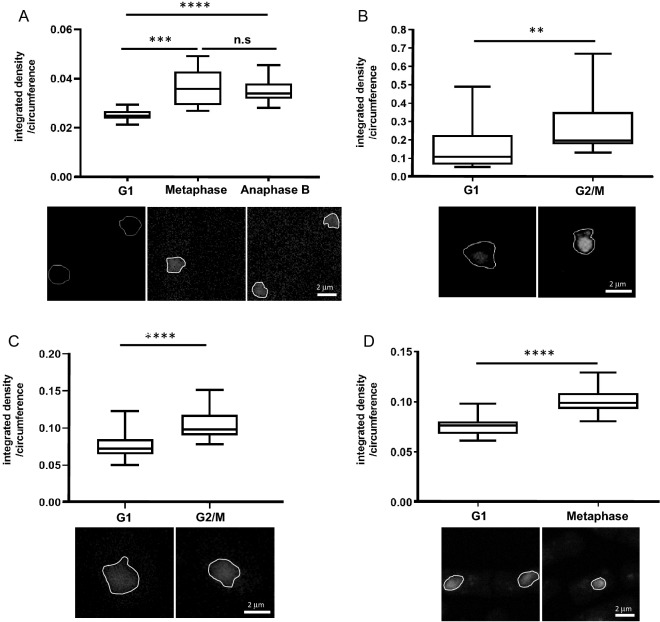
Measurement of chromosome compaction in live and fixed yeast. (**A**) Cells were grown to mid-log phase and analyzed via two-photon microscopy. Cells in G1/S, metaphase, and anaphase B were selected for live-cell analysis based on morphology and localization of the Cdc11-GFP (as shown in Supplementary Figure S1). 20 nuclei from each cell cycle phase were analyzed. **** *p* < 0.0001. n.s.—not significant. (**B**) *S. cerevisiae* cells were grown to mid-log phase and stained with Hoechst33342. The cell cycle phase was determined by cell morphology. Chromatin was analyzed via two-photon microscopy. 28 nuclei were analyzed from each cell cycle phase. ** *p* < 0.001. (**C**) *S. cerevisiae* cells were grown to mid-log phase, fixed, and stained with DAPI. The cell cycle phase was determined by cell morphology. Chromatin was analyzed via two-photon microscopy. 25 nuclei were analyzed from each cell cycle phase. **** *p* < 0.0001. (**D**) *S. pombe* cells were grown to mid-log phase, fixed, and stained with DAPI. The cell cycle phase was determined by cell morphology. Chromatin was analyzed via two-photon microscopy. 27 nuclei were analyzed from each cell cycle phase. **** *p* < 0.0001.

Chromatin compaction of cells in G1/S, metaphase, and anaphase B was analyzed by dividing the integrated density of the chromatin signal by the nucleus circumference (Fig. 1A)^16^. Importantly, the condensation level, measured by two-photon microscopy, is independent of nuclear size. This allows comparison between cells regardless of their nuclear dimensions, which can vary between cell cycle stages and species. Chromosomes in metaphase and anaphase B were 1.435- and 1.369-fold more condensed, respectively, than chromatin at G1/S (Fig. 1A). Of note, the condensation level of *S. pombe* is similar to the condensation level previously reported in *S. pombe* (1.53- to 1.84-fold)^15^ and *S. cerevisiae* (1.5- to 2-fold)^9,14,19^. These results demonstrate that determining chromatin compaction by two-photon microscopy can be applied to yeasts other than *S. cerevisiae*.

### Analyzing chromatin condensation in fixed cells

One of the limitations of the technique we describe is the need to achieve a precise focus on chromatin. Using live cells, this requirement is achieved using fluorescently-tagged histone proteins. However, fluorescent tagging is time-consuming, at best, and not possible at all in many non-model organisms. To overcome this limitation, we tested a different approach. *S. cerevisiae* cells, strain yIO-001, were grown to mid-log phase and stained with Hoechst33342. Condensation was then analyzed in G1- and G2/M-phase cells, which were selected based on their morphology. The fold-condensation between G1 and G2/M cells was1.617 (Fig. 1B), which was similar to the 1.393-fold change we detected in live cell analysis of cells expressing GFP-tagged histone H2B^16^.

We modified the two-photon microscopy method so we could study fixed cells by focusing on nuclear chromatin stained with DAPI. To explore the feasibility of this technique, we grew *S. cerevisiae* cells to mid-log phase and then fixed and stained them with DAPI (see “Materials and methods” section). We focused on the DAPI-stained nuclei and analyzed chromatin condensation by two-photon microscopy. To avoid collecting DAPI emission background, we performed the analysis with laser excitation at 768 nm, and emission was collected at 512 nm. Under these conditions, the change in chromatin condensation in *S. cerevisiae* fixed cells was 1.374-fold (Fig. 1C) between G1 and G2/M cells, which is similar to the 1.393-fold difference in live cells^16^. Next, we used the same conditions to analyze fixed *S. pombe*. In this experiment, we used cell morphology to differentiate between G1 and metaphase cells, which resulted in a 1.317-fold change, similar to the observed G1 to metaphase change in live cells (Fig. 1D). Thus, two-photon microscopy provides evidence of similar mitotic chromosome compaction in fixed as well as in live cells.

### The chromatin condensation level in yeast is a conserved property

**Figure 2:**
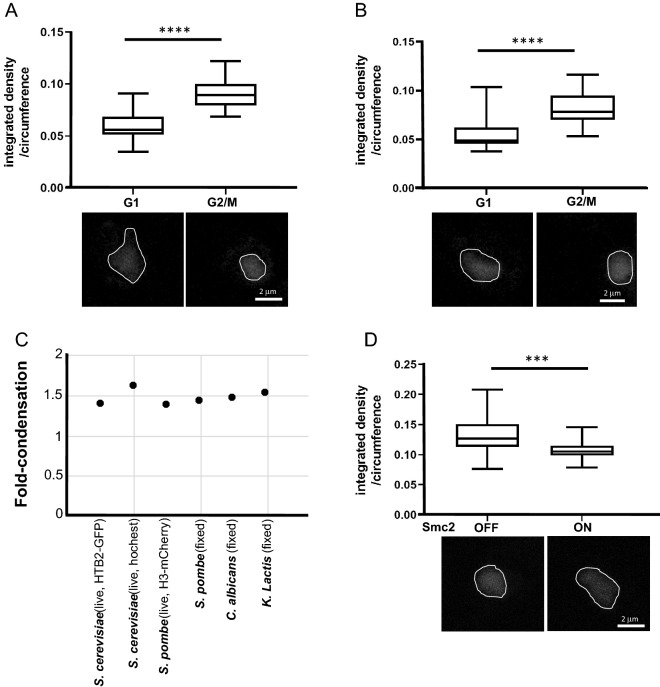
Analysis of chromosome compaction in different yeast cells. (**A**) *K. lactis* cells were grown, fixed, and stained with DAPI. 25 nuclei were analyzed from each cell cycle phase. **** *p* < 0.0001. (**B**) *C. albicans* cells were fixed and stained with DAPI. 30 nuclei were analyzed from each cell cycle phase. **** *p* < 0.0001. (**C**) Fold-compaction of chromatin in G2/M phase relative to G1 phase for yeast species is indicated. 25 nuclei were analyzed from each cell cycle phase. (**D**) *C. albicans* cells of strain Y30 (*Ptet-*SMC2) were grown to mid-log phase without and with doxycycline, fixed, and stained with DAPI. G2/M phase cells were selected based on morphology and analyzed via two-photon microscopy. 30 nuclei were analyzed from each condition. **** *p* < 0.0001.

Omitting the need to genetically manipulate strains for our study enabled our ability to analyze cell cycle-dependent chromatin condensation in a broader range of yeast species. Hence, we grew the industrial yeast *K. lactis* and the pathogenic yeast *C. albicans* to mid-log phase, fixed and stained the cells with DAPI, identified G1- or G2/M-phase cells based on their morphology and DAPI localization, and analyzed chromatin condensation with SGH microscopy (Supplementary Fig. S2). The chromatin compaction in the G2/M phase relative to the G1 phase was 1.526-fold in *K. lactis* and 1.469-fold in *C. albicans* (Fig. 2A, B). Since the condensation level measured by two-photon microscopy is independent of nuclear size, similar degrees of condensation in these yeast species suggest that the condensation level is an evolutionarily conserved property (Fig. 2C).

### Decondensation induced by depletion of the condensin subunit Smc2 in *C. albicans*

In eukaryotic cells, the condensin complex is crucial for mitotic chromosome condensation^3,4,11^. Accordingly, depletion of condensin in *S. cerevisiae* results in decondensation that can be detected by two-photon microscopy^16^. To ask if condensin defects affect chromatin condensation in another yeast, we used *C. albicans* strain Y30, in which expression of the condensin SMC2 subunit is expressed at levels higher than the native protein and is repressed if tetracycline or doxycycline is added.

SMC2 is a gene essential for cell viability. UMN7150 (wild-type) and Y30 (homozygous PTET-SMC2) cells were grown to saturation, and their growth was assessed. Stains grow equally well on YPD plates on which SMC2 expression is induced in Y30 cells. On YPD plates containing 50 μg/ml doxycycline, cell growth was 100–1000-fold slower than wild-type cells (Supplementary Fig. S4). This result suggests that SMC2 is not fully repressed in these cells.

Strain Y30 cells were grown to mid-log phase in the presence or absence of doxycycline and fixed. G2/M phase cells were selected based on their morphology and analyzed via two-photon microscopy. The degree of chromatin condensation in tetracycline-treated, Smc2-depleted, G2/M cells was ~ 1.22-fold less relative to that for untreated cells. Thus, as expected, based on the inferred function of condensin, reduced expression of SMC2 results in chromatin decondensation (Fig. 2D).

### Analysis of mitotic condensation in trypanosomes

**Figure 3:**
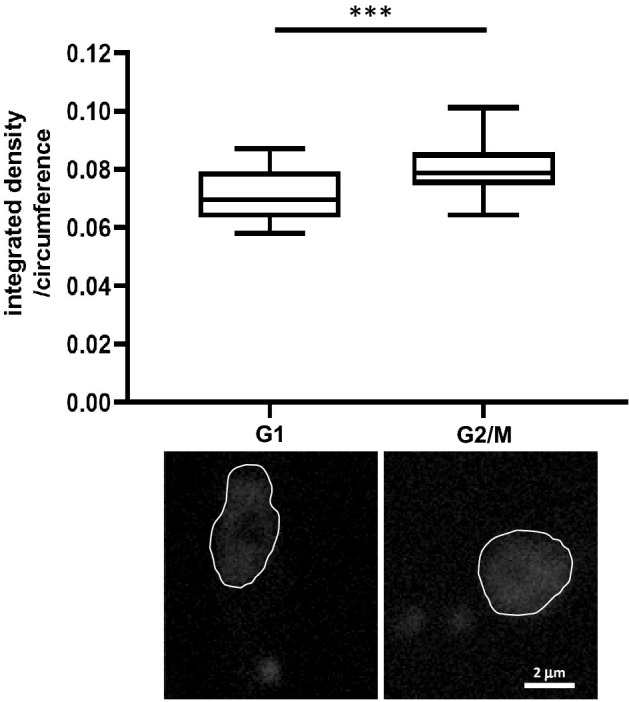
Analysis of chromosome compaction in *T. brucei*. 1 × 10^7^ cells were fixed, stained with DAPI, and analyzed via two-photon microscopy. The cell cycle stage of the cells was determined by the number of kinetoplasts and their localization with respect to the nucleus. 25 nuclei were analyzed from each cell cycle phase. *** *p* < 0.0003.

The ability to analyze chromatin condensation in fixed cells inspired us to conduct SHG analyses on other microorganisms, including protists. In particular, we studied *T. brucei brucei*, which is the unicellular eukaryotic non-human parasite, primarily of cattle and occasionally other animals. *T. brucei* cells contain a nucleus and a single mitochondrion (kinetoplast) and the cell-cycle state can be determined by kinetoplast duplication, which is synchronized with nuclear DNA replication and can be inferred by kinetoplast localization relative to the nucleus (^25^ and Supplementary Fig. S3). Through two-photon microscopy analysis of fixed *T. brucei* cells in both the G1 and G2/M phases of the cell cycle, we found that the integrated density/circumference of G2/M-phase cells was ~ 1.2-fold greater than G1-phase cells (Fig. 3). The difference in condensation levels between yeast and trypanosome may indicate a lower condensation in trypanosome. However, it may also not reflect the full compaction of the chromatin in mitosis, as discussed below.

### HCV induces decondensation in hepatoma cells

As larger chromosomes can be visualized directly, indirect fluorescence measurement is less critical for analyzing chromatin condensation in higher eukaryotic cells. Nonetheless, spectroscopic analysis of interphase chromatin in higher eukaryotes can offer a new approach to measuring subtle changes in chromatin organization induced by cellular factors or environmental conditions. We previously found that hepatitis C viral infection of human hepatoma cells is associated with the misregulation of cohesin in interphase^24^. As in yeasts, in mammalian cells, cohesin is a key factor in interphase chromatin organization^26–29^. Thus, we hypothesized that HCV-dependent misregulation of cohesin might lead to changes in chromatin organization in infected cells.

**Figure 4:**
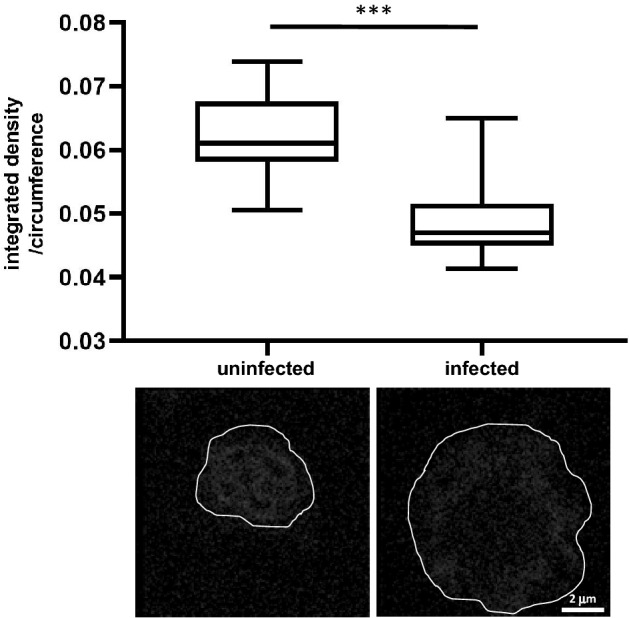
HCV decondense interphase chromatin in infected cells. Huh-7.5 cells were grown untreated or infected with HCV. Cells were processed for two-photon microscopy and analyzed. 30 nuclei were analyzed from each cell cycle phase. *** *p* < 0.0001.

By using two-photon microscopy to analyze hepatoma Huh-7.5 cells that were, or were not, infected with HCV, we found that nearly 100% of the infected cells were HCV-positive at fourteen days post-infection. These cells were resuspended by trypsinization, fixed, and stained with DAPI. The integrated density/circumference of the HCV-infected cells was ~ 1.3-fold less than uninfected cells (Fig. 4). Thus, interphase chromatin in HCV-infected cells is ~ 30% less condensed than in uninfected cells. These results are consistent with the massive upregulation of gene expression following infection, resulting in chromatin reorganization in interphase^24,30^.

## Discussion

Here, we analyzed the degree of chromatin condensation in mitosis relative to interphase using two-photon microscopy applied to a broad range of live and fixed eukaryotic cells, including other types of yeast, protozoa, and uninfected as well as virus-infected mammalian cells. This illustrates the utility of two-photon microscopy as a general technique for analyzing chromatin organization in cell types ranging from simple unicellular eukaryotes to mammalian cells. The method is sensitive enough to detect changes of 1.2-fold in condensing mutants and can be applied to non-model organisms for a better understanding of mitotic chromosome dynamics. Importantly, in the unicellular eukaryotes tested, the level of mitotic condensation relative to interphase chromatin is similar, ranging in this study from 1.2- to 1.6-fold in comparison with interphase chromatin.

The four types of yeast analyzed here (*S. cerevisiae*, *S. pombe*, *K. lactis*, and *C. albicans*) belong to different phylogenetic branches of the ascomycete phylum of fungi and have diverse genome sizes (12–18 Mb) and numbers of chromosomes (3 haploid chromosomes in *S. pombe* to 6, 8, and 16 chromosomes in *K. lactis*, *C. albicans*, and *S. cerevisiae*, respectively) (Supplementary Table 2). However, relative to interphase chromatin, the mitotic condensation level in all of the yeast cells was similar, ranging from 1.393- to 1.526-fold (*S. cerevisiae* and *K. lactis*, respectively). These findings suggest that the degree of chromosome condensation in yeast is a conserved property.

When we depleted condensin *in* mitotic *C. albicans*, chromatin decondensed by ~ 1.3-fold in comparison with interphase chromatin^16^. This was similar to the fold-change decondensation seen when condensin was depleted in *S. cerevisiae*. The differences between these measurements are likely due to the experimental approaches that resulted in different levels of residual condensin in cells. Depletion of the *S. cerevisiae* BRN1 condensin subunit was accomplished using an auxin-induced degron that leads to undetectable levels of the protein in Western blot analysis. By contrast, repression of the PTET promoter that was used to suppress *C. albicans SMC2* proved to be leaky, with cells containing residual levels of condensin that support partial organization of mitotic chromosomes.

We also analyzed chromatin condensation in a protozoan, *T. brucei* and found the G2/M phase to be ~ 1.2-fold more condensed than the G1 phase. While the degree of condensation appeared to be somewhat lower for *T. brucei* than for yeast, we suspect that measurements of 1.2-fold in *T. brucei* were biased by two explanations that are not mutually exclusive. First, it is more difficult to determine the cell cycle phase in trypanosomes than in yeast because early and late G2 cells cannot be differentiated based on kinetoplast localization. As a consequence, we believe that the sample of G2/M cells may have been biased toward early G2 and, thus, preceded significant mitotic condensation, which begins primarily in late G2 and during the entry into mitosis. Second, the *T. brucei* genome is highly complex compared to yeast genomes, being composed of 120 mitotically stable chromosomes, which are divided into three size classes: 11 pairs of megabase chromosomes (0.9–5.7 Mb), 2–4 pairs of intermediate chromosomes (300–900 kb), and ~ 100 minichromosomes (50–100 kb)^31^. We cannot exclude the possibility that different chromosomal size classes may differ in their condensation levels and interfere with the homogeneity of the measurement. One or both of these possibilities could have increased the heterogeneity of the analysis, which was reflected in a reduced fold-change in the mitotic condensation level relative to interphase. This similar degree of mitotic condensation in unicellular eukaryotes suggests that low-fold condensation is sufficient for the proper segregation of these relatively small chromosomes.

HCV, an RNA virus that infects hepatoma cells, is associated with hepatic fibrosis and the development of hepatocellular carcinoma. While the HCV life cycle ensues in the host cell’s cytoplasm, HCV affects host nuclear gene expression. Here, we found that HCV induces decompaction of interphase chromatin by ~ 0.78-fold, a degree of change that is consistent with virally-induced transcriptional changes. Furthermore, HCV infection affects cohesin localization at gene promoters^24,30^, leading to the hypothesis that altered cohesin localization may be associated with the reorganization of the host genome. Cohesin, together with CTCF plays a major role in the formation of topologically associated domains (TADs), as well as in intra-TAD interactions. In addition, recent studies have found that condensin regulates gene expression^32,33^. The chromatin decondensation that accompanies HCV infection, as seen here, is consistent with the idea that HCV-induced changes in cohesin localization are due to major changes in TAD formation and inter-TAD interaction in the host cell genome which would most likely alter chromatin structure.

In conclusion, two-photon microscopy is an excellent approach for studying chromatin organization in a broad range of cell types and biological conditions. In this work, we utilized it to examine the relation between chromatin condensation levels and genome stability and dynamics. It is likely to be useful for the study of chromatin changes in non-model microorganisms to explore their response to different environmental conditions such as temperature, salt, or heavy metals.

## Supplementary Information

Supplementary Information 1.

Supplementary Information 2.

Supplementary Information 3.

Supplementary Information 4.

Supplementary Information 5.

Supplementary Information 6.

## Data Availability

The datasets used and/or analyzed during the current study are available from the corresponding author upon reasonable request.
