# Supplementary material for: Fold-change of chromatin condensation in yeast is a conserved property
Source: Sci Rep. 2022 Oct 17;12:17393. doi: 10.1038/s41598-022-22340-8 (PMC9576780; doi:10.1038/s41598-022-22340-8)
Supplement: Supplementary file 1 — Supplementary Information 1. [file 41598_2022_22340_MOESM1_ESM.pdf]

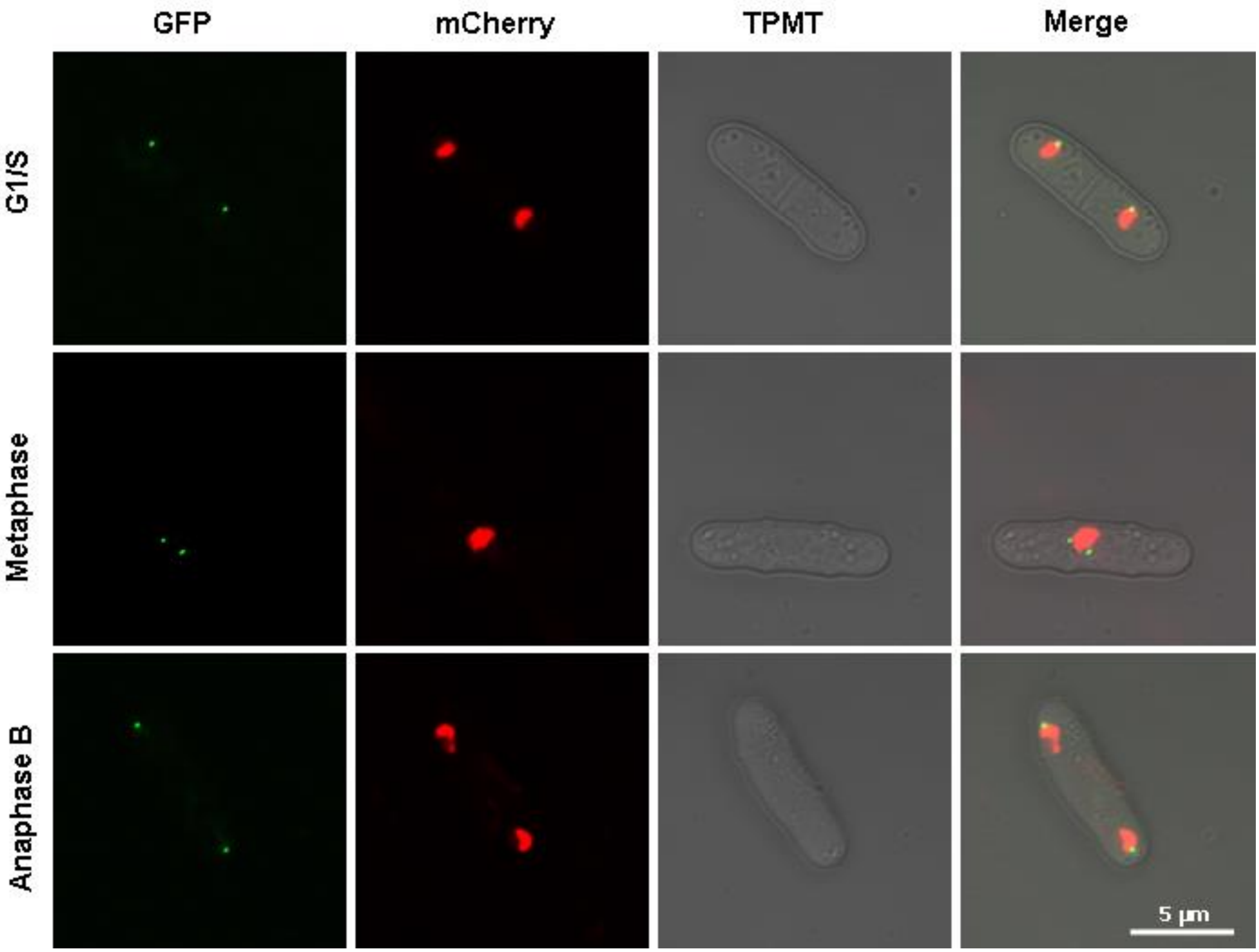

Supplementary Figure S1. Determination of the cell cycle phase in *S. pombe*. Cells contained the centrosome protein Cdc11-GFP and histone Hht1-mCherry. In the G1 phase, cells are separated and the septum is complete, and Cdc11 is located at the periphery of the nucleus. In metaphase, the centrosome is duplicated and can be seen as two separate dots at the periphery of the nucleus. In anaphase B, the genome is segregated, but cell division is not completed as the septum is absent.
