# Supplementary material for: Fold-change of chromatin condensation in yeast is a conserved property
Source: Sci Rep. 2022 Oct 17;12:17393. doi: 10.1038/s41598-022-22340-8 (PMC9576780; doi:10.1038/s41598-022-22340-8)
Supplement: Supplementary file 2 — Supplementary Information 2. [file 41598_2022_22340_MOESM2_ESM.pdf]

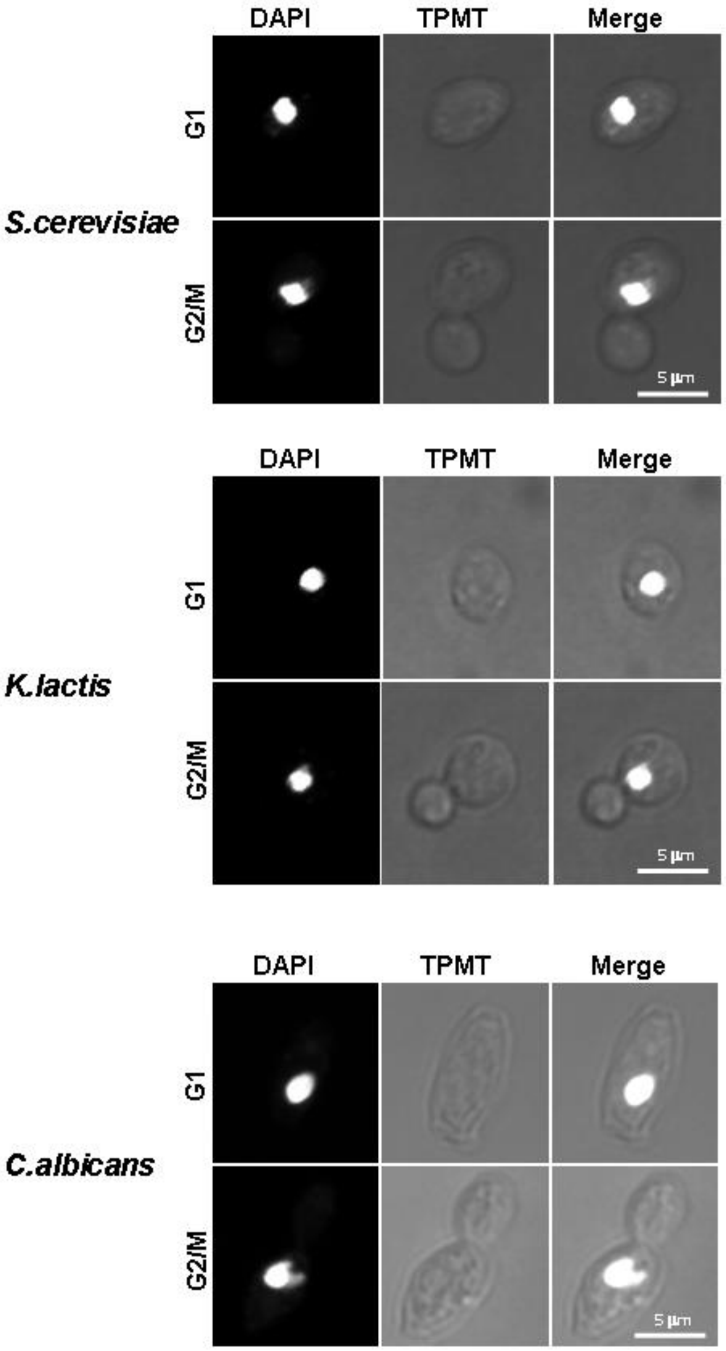

Supplementary Figure S2. Determination of the cell cycle phase in budding yeast. The G1 and G2/M phases of the cell cycle of budding yeast were determined based on the morphology of the cells. G1 cells are round with no bud. G2/M cells are two, full-sized cells with a single nucleus located near the bud site. *S.cerevisiae, K. lactis*, and *C. albicans* cells are shown.
