# Supplementary material for: Fold-change of chromatin condensation in yeast is a conserved property
Source: Sci Rep. 2022 Oct 17;12:17393. doi: 10.1038/s41598-022-22340-8 (PMC9576780; doi:10.1038/s41598-022-22340-8)
Supplement: Supplementary file 3 — Supplementary Information 3. [file 41598_2022_22340_MOESM3_ESM.pdf]

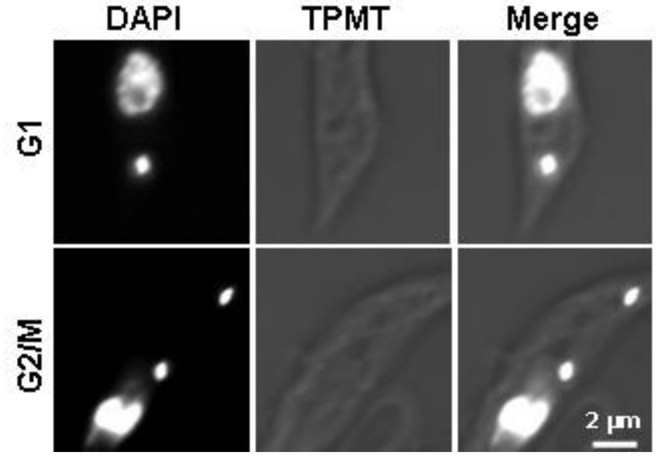

Supplementary Figure S3. Determination of the cell cycle phase in *T. brucei*. The G1 and G2/M phases of the cell cycle of *T. brucei* cells were determined based on the presence of one or two kinetoplasts in cells with a single nucleus. The kinetoplast DNA is stained with DAPI.
