# Supplementary material for: Fold-change of chromatin condensation in yeast is a conserved property
Source: Sci Rep. 2022 Oct 17;12:17393. doi: 10.1038/s41598-022-22340-8 (PMC9576780; doi:10.1038/s41598-022-22340-8)
Supplement: Supplementary file 4 — Supplementary Information 4. [file 41598_2022_22340_MOESM4_ESM.pdf]

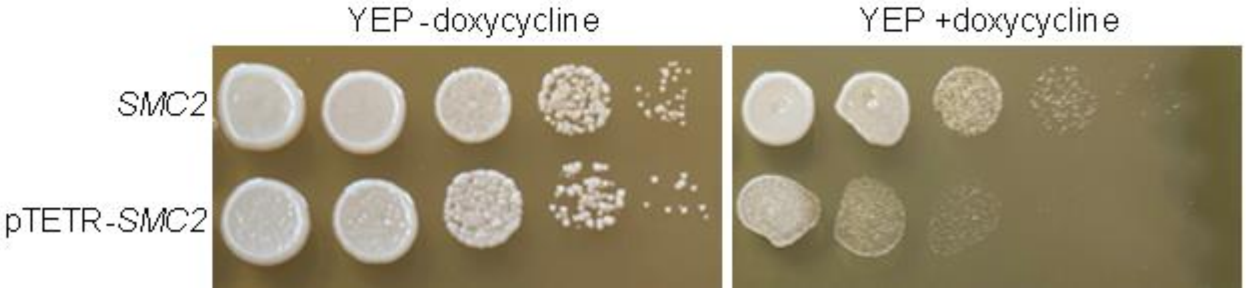

Supplementary Figure S4. Depletion of Smc2 in C. albicans. A. Strains UMN7150 (wild type Y30 (homozygous Ptet-SMC2) were grown in YPD to saturation, serially diluted and plated on YPD plates without or with 50 μg/ml doxycycline. Plates were incubated for one day at 30°C.
