# Supplementary material for: Fold-change of chromatin condensation in yeast is a conserved property
Source: Sci Rep. 2022 Oct 17;12:17393. doi: 10.1038/s41598-022-22340-8 (PMC9576780; doi:10.1038/s41598-022-22340-8)
Supplement: Supplementary file 5 — Supplementary Information 5. [file 41598_2022_22340_MOESM5_ESM.pdf]

Supplementary Table S1. Yeast Strains

Spices	Strain	Genotype	Source
S. cerevisiae	yIO-001	*MATa pep4Δ::g418::TRP1 trp1-1 leu2-3,112 ura3-52 his3-11,15 bar1 GAL+*	IO
S. pombe	yIO984	*LY5628:h- leu1-32 ura4D ade6-210 cdc11-GFP-NatR hht1-mCherry-HygroR*	A gift from Pascal Bernard to IO
K.lactis	7B520	ura3-1, his2-2, trp1.	A gift from Yehuda Tzfati to IO
C. albicans	UMN7150	*BWP1, HHF2-GFP*	JB
C. albicans	Y30	*HAH2-Ptet-SMC2/HIS1-Ptet-SMC2 HHF1-GFP-URA3/HHF1 ADH1/adh1::PTDH3-tTA SN76*	JB
T. brucei brucei	29-13		JS
Huh-7.5	N/A		a gift from Charles Rice to MGT
