# Supplementary material for: Fold-change of chromatin condensation in yeast is a conserved property
Source: Sci Rep. 2022 Oct 17;12:17393. doi: 10.1038/s41598-022-22340-8 (PMC9576780; doi:10.1038/s41598-022-22340-8)
Supplement: Supplementary file 6 — Supplementary Information 6. [file 41598_2022_22340_MOESM6_ESM.pdf]

Supplamentry Table 2. Genome properties of yeast species

Yeast	Phylogenetics	Genome size (Mb)	Chromosome number
Saccharomyces cerevisiae	Saccharomycetaceae	12	16
Kluyveromyces lactis	Saccharomycetaceae	14	6
Schizosaccharomyces pombe	Taphrinomycotina	10.6	3
Candida albicans	Debaryomycetaceae-Metschnikowiaceae	14-18	8
